# Accelerated Molecular Dynamics Simulation for Helical Proteins Folding in Explicit Water

**DOI:** 10.3389/fchem.2019.00540

**Published:** 2019-08-06

**Authors:** Lili Duan, Xiaona Guo, Yalong Cong, Guoqiang Feng, Yuchen Li, John Z. H. Zhang

**Affiliations:** ^1^School of Physics and Electronics, Shandong Normal University, Jinan, China; ^2^Shanghai Engineering Research Center of Molecular Therapeutics and New Drug Development, School of Chemistry and Molecular Engineering, East China Normal University, Shanghai, China; ^3^NYU-ECNU Center for Computational Chemistry at NYU Shanghai, Shanghai, China; ^4^Department of Chemistry, New York University, New York, NY, United States

**Keywords:** accelerated molecular dynamics simulation, helical protein, protein folding, explicit water, free energy landscape

## Abstract

In this study, we examined the folding processes of eight helical proteins (2I9M, TC5B, 1WN8, 1V4Z, 1HO2, 1HLL, 2KFE, and 1YYB) at room temperature using the explicit solvent model under the AMBER14SB force field with the accelerated molecular dynamics (AMD) and traditional molecular dynamics (MD), respectively. We analyzed and compared the simulation results obtained by these two methods based on several aspects, such as root mean square deviation (RMSD), native contacts, cluster analysis, folding snapshots, free energy landscape, and the evolution of the radius of gyration, which showed that these eight proteins were successfully and consistently folded into the corresponding native structures by AMD simulations carried out at room temperature. In addition, the folding occurred in the range of 40~180 ns after starting from the linear structures of the eight proteins at 300 K. By contrast, these stable folding structures were not found when the traditional molecular dynamics (MD) simulation was used. At the same time, the influence of high temperatures (350, 400, and 450 K) is also further investigated. Study found that the simulation efficiency of AMD is higher than that of MD simulations, regardless of the temperature. Of these temperatures, 300 K is the most suitable temperature for protein folding for all systems. To further investigate the efficiency of AMD, another trajectory was simulated for eight proteins with the same linear structure but different random seeds at 300 K. Both AMD trajectories reached the correct folded structures. Our result clearly shows that AMD simulation are a highly efficient and reliable method for the study of protein folding.

## Introduction

Protein misfolding can cause a series of diseases, such as Parkinson's disease, Alzheimer's disease, mad cow disease, and Gusher's disease (Dobson, 1999; Slepoy et al., 2001; Muchowski, 2002; Cohen and Kelly, 2003; Chiti and Dobson, 2006). The function of a protein is determined by its three-dimensional structure and dynamics. Therefore, it is of great significance to understand the structural characteristics and dynamic behavior of proteins in theoretical and experimental studies, in particular for the process of folding from the extended state into the native structure of the protein. It is difficult to obtain microscopic information for the folding process and mechanism in atomic details using experimental techniques. With the increase in computing power, all-atom molecular dynamics (MD) simulations have become a popular tool for the studies of protein folding for many researchers (Duan and Kollman, 1998; Brooks, 2002; Daggett, 2002; Eleftheriou et al., 2006; Yoda et al., 2007; Hua et al., 2008; Nelson and Grishin, 2008; Lindorff-Larsen et al., 2011; Nguyen et al., 2014; Weber et al., 2014; Li et al., 2015; Bernhardt et al., 2016; Perez et al., 2016; Schwantes et al., 2016). In principle, all desired information regarding the kinetics and thermodynamics of proteins can be obtained by MD simulation (Straatsma and Berendsen, 1988; Lindorff-Larsen et al., 2012; Meher and Wang, 2012; Chen et al., 2013, 2015; Tzoupis et al., 2013; Brogan et al., 2014). However, many studies of direct protein folding are limited to small proteins due to the various difficulties in the current MD simulations (Ferrara et al., 2000; Yang et al., 2009; Shaw et al., 2010). The main bottleneck is the time scale of the MD simulation. Some biological important processes such as enzyme catalysis, protein-ligand binding, signal transduction, and allosteric regulation require the time-scales ranging from microsecond to millisecond (Markwick and McCammon, 2011).

Using massive parallel clusters, the MD simulation time can reach to the millisecond range. Unfortunately, not all researchers have access to such large-scale supercomputing resources. The development of an algorithm to solve the time scale problem and enhance the conformational sampling in finite time provides a possible shortcut for reaching long time scales; this method also fully retains the atomistic representation of the system (Berne and Straub, 1997; Elber, 2005). To overcome kinetic trapping and achieve thorough sampling of the conformational space of proteins, several methods including umbrella sampling (Torrie and Valleau, 1977), multicanonical algorithms (Berg and Neuhaus, 1992), simulated tempering (Marinari and Parisi, 1992), transition path sampling (Bolhuis et al., 2002), targeted molecular dynamics (Schlitter et al., 1994; Ma et al., 2000), replica exchange method molecular dynamics (REMD also known as parallel tempering) (Hukushima and Nemoto, 1996; Hansmann, 1997; Sugita and Okamoto, 1999; García and Sanbonmatsu, 2002), and accelerated molecular dynamics (AMD) (Hamelberg et al., 2004) have been developed. Among these, the AMD method has become a popular method in protein folding simulations.

The AMD method firstly proposed by Voter (1997a,b) and recently improved by the group of J. A. McCammon (Hamelberg et al., 2004) is an efficient method for enhancing conformational space sampling. The most important advantage of AMD relative to the other aforementioned biased free energy calculations mentioned above is that AMD does not require any predefined reaction coordinates. This method reduces the energy barrier by increasing a non-negative boost potential and increases the transition probability between the different low-energy states, while, the potential energy landscape still retains important details (Hamelberg et al., 2004; Hamelberg and McCammon, 2005; Markwick et al., 2007; Bucher et al., 2011). The AMD method has been successfully used in simulation of many proteins. These proteins include alanine dipeptide (Miao et al., 2014b), a silk-like polypeptide (Zhao et al., 2017), fast-folding proteins (Miao et al., 2015), G-protein coupled receptors (Miao et al., 2014a), bovine pancreatic trypsin inhibitor (BPT1) (Pierce et al., 2012), streptavidin-biotin complex (Song et al., 2015), α-1-Antitrypsin (Andersen et al., 2017), MSI-594 (Mukherjee et al., 2017), and insulin (Nejad and Urbassek, 2018).

Here, we apply AMD and traditional MD methods to study the folding of eight proteins (2I9M, TC5B, 1WN8, 1V4Z, 1HO2, 1HLL, 2KFE, and1YYB) at 300 K. The explicit solvent model is used the above simulation. Although this model requires more computational time than the implicit solvent model, it is more accurate for the exploration of the folding process (Duan et al., 2012). Among the proteins investigated in the present work, TC5B proteins have been studied as a reference system for many groups due to their relatively small size and fast folding kinetics (Simmerling et al., 2002). A folding simulation study of TC5B protein using the implicit solvent model (Generalized Born: GB) with AMBER99 force field at 325 K performed by Simmerling et al. (2002) found the similarity between the NMR models and the low energy structure. Duan et al. used different combinations of force fields and solvation models to simulate the thermodynamics of folding and unfolding of TC5B using the REMD method (Duan et al., 2010). They found that the thermodynamic properties of TC5B are very sensitive to the specific version of the solvation model and the force field used. Shao et al. performed the integrated-tempering-sampling molecular dynamics (ITS-MD) simulation under three force fields (FF03, FF99SB, and FF96) and the GB implicit solvent model (Shao et al., 2012). They concluded that the combination of the FF03 force field and ITS-MD provides thermodynamic results in good agreement with the experimental values. Zhou et al. employed the REMD method with the residue-specific force field 2 (RSFF2) to investigate the thermodynamics and folding mechanism of five TC5B variants (Zhou et al., 2015). They found that different variants have the same major folding pathway and obtained results consistent with the experiment data. To date, there is still a lack of AMD simulation studies of the folding of TC5B under the explicit solvent model.

For evaluate the efficiency and accuracy of AMD simulations, we compare the results obtained by AMD and MD simulation for root mean square deviation (RMSD), native contact, cluster analysis, the folding process, free energy landscape, and the radius of gyration. The obtained results clearly show that all eight proteins successfully fold into the corresponding native structures within 180 ns using the AMD method; however, they all fail to fold in traditional MD simulation. The same conclusion is again drawn from the multiple-trajectory AMD simulation results. In addition, it is still unclear that the effect of the temperatures on the helical protein folding at the atomic level, and high temperature is helpful for accelerating the sampling in the phase space. Therefore, the effect of different temperatures (350, 400, and 450 K) for the eight systems is also further investigated in this study.

## Theoretical Methods

### Accelerated Molecular Dynamics

Generally, the potential energy barriers are always very high during the MD simulation for protein folding, so that in traditional MD simulation, the systems are usually trapped in local minimum. The exchange rate between the low energy conformation states of the system is very low. To increase the rates, the AMD method based on traditional MD was developed by the McCammon group (Hamelberg et al., 2004). The primary difference between AMD and traditional MD is that the AMD adds a robust bias potential energy to the actual potential, so that the heights of the local barriers are reduced by using the AMD method. The parameters of AMD and MD of each system are the same, except that a biased potential energy is added to the real potential in the AMD method. The total simulation time of AMD and MD runs in each system is the same. However, prior to the AMD simulation, a 1 ns traditional MD simulation is carried out to obtain the initial parameters that are used to define the added potential energy.

The four parameters are obtained from 1 ns traditional MD (*E*_*D*_, α_*D*_, *E*_*P*_, α_*P*_) by the following equations:

(1) $$\begin{array}{r} V^{{\;}^{\prime }}\left(r\right)=V\left(r\right)+\Delta V\left(r\right) \end{array}$$

(2) $$\begin{array}{r} \Delta V\left(r\right)=\frac{{\left(E_{P-}V\left(r\right)\right)}^{2}}{\left(\alpha_{P}+E_{P}-V\left(r\right)\right)}+\frac{{\left(E_{D-}V_{D}\left(r\right)\right)}^{2}}{\left(\alpha_{D}+E_{D}-V_{D}\left(r\right)\right)} \end{array}$$

(3) $$\begin{array}{r} E_{D}=E_{D^{\prime }}+a_{1}N_{res}/5 \end{array}$$

(4) $$\begin{array}{r} \alpha_{D}=a_{1}\times N_{res}/5 \end{array}$$

(5) $$\begin{array}{r} E_{P}=E_{P^{\prime }}+a_{2}N_{atom} \end{array}$$

(6) $$\begin{array}{r} \alpha_{P}=a_{2}\times N_{atom} \end{array}$$

Here, *V*(*r*) is the actual potential, *V*_*D*_(*r*) is the actual dihedral torsion potential, and *V* is the added potential energy. *N*_*res*_ and *N*_*atom*_ are the number of residues and the total number of atoms per protein, respectively. $E_{P^{\prime }}$ and $E_{D^{\prime }}$ are the average potential energy and the dihedral potential energy, respectively, obtained from the 1 ns traditional molecular dynamics simulation. *E*_*P*_ and *E*_*D*_ are the threshold energies, which are used to run AMD simulation. They are calculated by Equations (3) and (5), in which *a*_1_ and *a*_2_ are 3.5 and 0.2 kcal/mol from the Amber12 manual, respectively. All of the simulation parameters are shown in Table S1 under different temperatures in the Supporting Information. Here, *E*_*D*_, α_*D*_, *E*_*P*_, and α_*P*_ are given in kcal/mol.

### MD Simulation

The initial linear structures of the eight helix proteins are generated from the Protein Data Bank (PDB). The PDB entries are 2I9M, 1L2Y, 1WN8, 1V4Z, 1HO2, 1HLL, 2KFE, and 1YYB. They are randomly selected from the PDB database based on the number of residues and helix structure. Considering that too large proteins are not easy to fold, the range of amino acid numbers is set from 17 to 32. The number of residues in the eight systems is 17, 20, 22, 17, 20, 32, 24, and 26, respectively. All of the missing hydrogen atoms were built from Leap module in AMBER12 with the FF14SB force field (Maier et al., 2015). Each of the eight systems was placed in a TIP3P (Jorgensen et al., 1983) water box, and the distance from the surface of the water box to all of the atoms of the solute is set to 10 Å. Counterions were added to each system to neutralize the charge. Energy minimization was carried out using the steepest descent method followed by the conjugate gradient minimization until convergence. Then, these systems were heated up from 0 to 300, 350, 400, and 450 K in 300 ps, respectively. And the Langevin (Pastor et al., 1988) dynamics with the collision frequency of 1.0 ps^−1^ were used to adjust the temperature. The SHAKE algorithm (Ryckaert et al., 1977) restrains all bonds involving hydrogen atoms. The long-range electrostatic interaction was calculated by the PME (particle mesh Ewald summation) (Darden et al., 1993). Then, the AMD and MD simulations were carried out. First, for each of the eight systems, 1 ns MD simulation was performed to obtain these parameters needed for AMD, and then each system was simulated with AMD and MD at the same time. The simulation times of AMD and MD for these proteins with the explicit solvent model were 51, 146, 146, 81, 196, 86, 61, and 86 ns, respectively.

## Results and Discussion

### Root Mean Square Deviation (RMSD)

**Figure 1:**
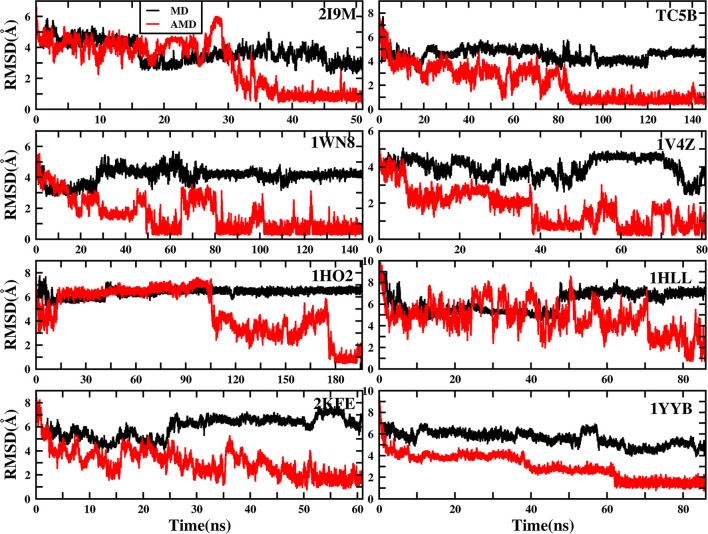
RMSD of helix structure of 2I9M, TC5B, 1WN8, 1V4Z, 1HO2, 1HLL, 2KFE, and 1YYB simulated by AMD and MD under the explicit solvent model at 300 K.

**Figure 2:**
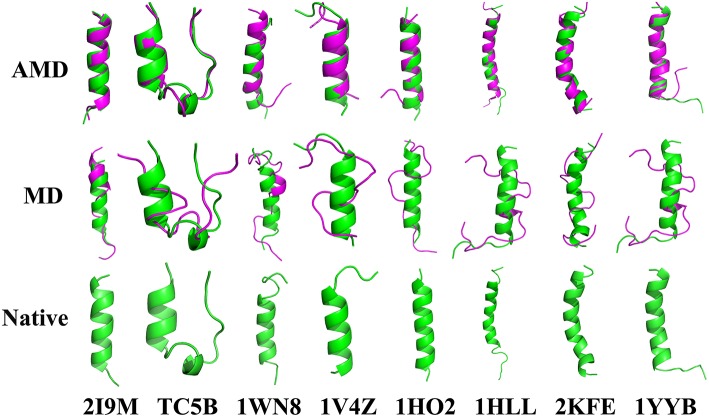
Folding of 2I9M, TC5B, 1WN8, 1V4Z, 1HO2, 1HLL, 2KFE, and 1YYB simulated by AMD and MD at 300 K: the structures with the lowest RMSD from the simulation of those system. The simulated structure (pink) are overlaid with the native structure (green).

To evaluate the quality of protein folding simulations, the variations of RMSDs of the helix regions with the simulation time in comparison to the corresponding native structures for the eight proteins obtained using the AMD and MD simulations at 300 K are calculated and shown in Figure 1 and Table S2 in the Supporting Information. It is observed that in all of the simulations of these eight systems, the overall fluctuation of RMSD generated by MD is larger than that obtained from the AMD simulations. These eight proteins fail to fold into native structures in the MD simulations. By contrast, the AMD simulation successfully folds the eight proteins with the final RMSD values of 0.77, 0.65, 0.66, 0.98, 1.10, 1.93, 1.49, and 1.51 Å vs. 2.69, 4.72, 4.19, 3.53, 6.56, 7.17, 6.13, and 4.941 Å obtained by MD simulations. The structure with the lowest RMSD obtained from the AMD simulation and MD simulation are shown in Figure 2. Clearly, these structures obtained in the AMD simulations match the native structures quite well, suggesting that they have successfully reached their native states.

The time of the folding process using the AMD method can also be deduced from Figure 1 and Table S2, and it is observed that beginning formation time of the first helical structures are 5.60, 10.00, 4.64, 6.20, 2.00, 8.00, 3.40, and 36.4 ns for the eight proteins, respectively. Their RMSDs drop from the initial values of 6.92, 8.36, 7.25, 6.11, 8.79, 11.68, 9.98, and 10.80 to 4.37, 3.96, 4.20, 3.80, 4.47, 4.21, 4.04, and 3.76 Å, respectively. Then these proteins start to form stable helical structures in ~40.20, 90.00, 86.00, 43.00, 180.00, 71.60, 55.20, and 64.20 ns with the RMSD values fluctuating ~0.70, 1.30, 0.60, 1.65, 1.20, 2.50, 2.10, and 1.20 Å, respectively. This indicates that these AMD simulations have converged and the proteins folding were fully completed.

### Native Contact

**Figure 3:**
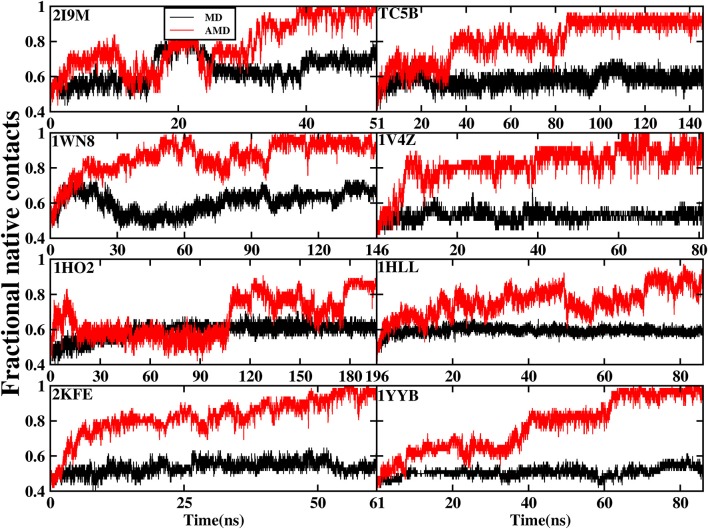
The percentage of native contacts during AMD simulation and MD simulation at 300 K as a function of time for 2I9M, TC5B, 1WN8, 1V4Z, 1HO2, 1HLL, 2KFE, and 1YYB, respectively.

The formation of native contact in the simulations is an important indicator for the evaluation of the folding process. In this analysis, native contact is defined as the distance of two C_α_ from non-adjacent residues of <7.0 Å. The fractional native contact is the number of the native contacts formed in the MD simulation divided by the number of native contacts in the native structure. The fractional native contact values obtained from AMD simulations are higher than those from the MD simulations at 300 K consistently at the same time in all of the systems shown in Figure 3. In the AMD simulations, the fractional native contact values reach the highest level close to 100% for 2I9M, TC5B, 1WN8, 1V4Z, 2KFE, and 1YYB and then remain relatively stable and fluctuated between 80 and 100%. By contrast, the fractional native contact values are relatively low in the MD simulations, fluctuating between 40 and 65% for most of the proteins, with the maximum value of only 70%. In the 1HO2 and 1HLL systems, the native contact fraction reaches ~80 and 90% and fluctuates between 70 and 90% during the AMD simulation, which is still higher than the corresponding values obtained from MD simulation. The above analysis indicates that these structures obtained in the AMD simulations are close to the native structures of the proteins.

Figure 3 provides further information about the native contact fractions for the eight systems examined in our work using AMD and MD simulations. The initial values of the fractional native contact for the AMD and MD simulation are the same, and are as follows: 30, 43, 36, 47, 35, 34, 38, and 41% for the eight studied systems, respectively. In AMD simulations, the native contact fractions of the eight systems increase to ~100, 95, 100, 100, 80, 80, 100, and 100% at 40, 90, 100, 60, 180, 70, 50, and 65 ns, respectively. On the other hand, the native contact fractions for the eight systems in MD simulations only increase to 60, 60, 60, 50, 60, 60, 50, and 50%. It is clear from Figure 3 that the fractional native contact obtained by AMD simulations is much higher than that in MD simulations during the subsequent simulation times with the fluctuation in the 85~100% range. These data clearly demonstrate that the linear structure is successfully folded into the native structure or approximately native structure in AMD simulations, while the folding fail in MD simulations.

### Cluster Analysis

**Figure 4:**
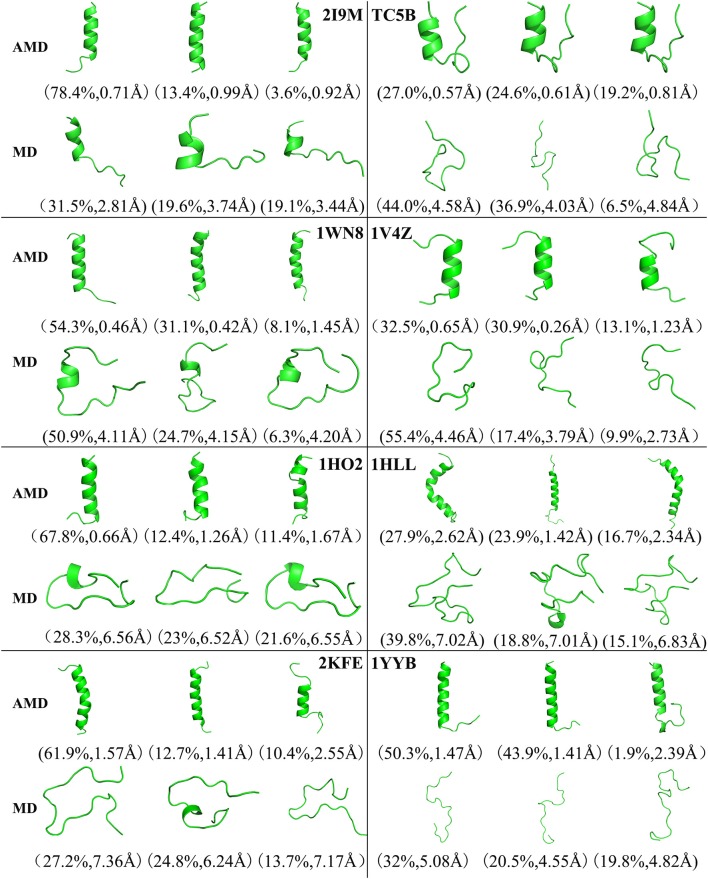
Representative structures of 2I9M, TC5B, 1WN8, 1V4Z, 1HO2, 1HLL, 2KFE, and 1YYB conformations selected from the top three occupied clusters using AMD simulation (top) and MD simulation (low) at 300 K. The population of clusters and the backbone RMSD of the cluster centers are indicated.

Figure 4 shows the representative structures of the top three occupied clusters for the eight studied proteins during the simulation process after relatively stable structure are achieved at 300 K. Those clusters have the highest population of 78.4, 27.0, 54.3, 32.5, 67.8, 27.9, 61.9, and 50.3% with the RMSD values of 0.71, 0.57, 0.46, 0.65, 0.66, 2.62, 1.57, and 1.47 Å in AMD simulations, in agreement with their corresponding native structures. For comparison, in the MD simulations, the clusters with the highest occupation have the RMSD values of 2.81, 4.58, 4.11, 4.46, 6.56, 7.02, 7.36, and 5.08 Å, respectively, and they are much higher than the values obtained from AMD simulations. A further analysis of the clusters obtained from AMD simulations shows that the proteins with the highest occupancy for 2I9M, TC5B, 1HO2, 2KFE, and 1YYB have the lowest RMSD values in all of the structures. Although the cluster with the highest occupancy for 1WN8, 1V4Z, and 1HLL do not show the lowest RMSD values, the second most occupied cluster of these systems have occupancies of 31.1, 30.9, and 23.9% with the RMSD values of 0.42, 0.26, and 1.42 Å, and both of the top two clusters of these systems are found in their native states. The above analysis suggests that the first three occupied clusters obtained with AMD simulations of the eight systems are in the native state or the native-like state, but the clusters in the MD simulations are not in their native states.

### Protein Folding Process

**Figure 5:**
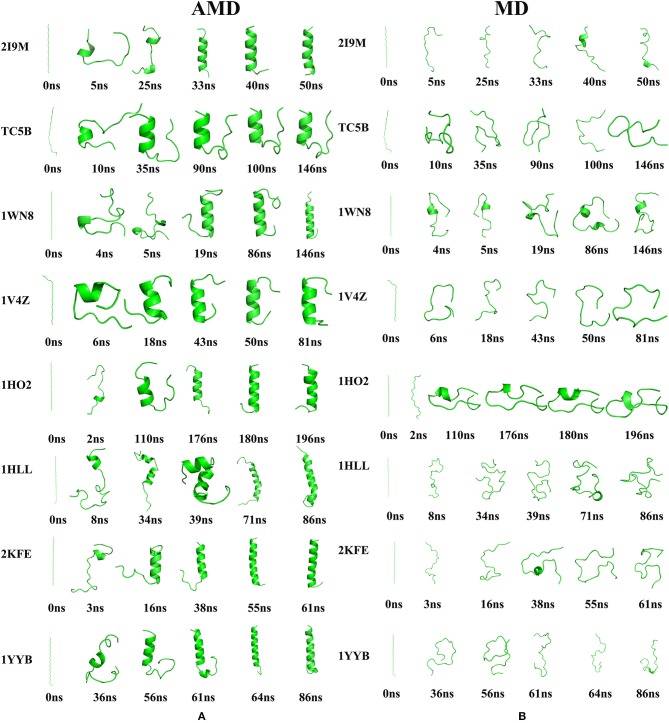
The evolution of the eight proteins **(A)** Snapshots of the intermediate conformation of 2I9M, TC5B, 1WN8, 1V4Z, 1HO2, 1HLL, 2KFE, and 1YYB at various simulation times using AMD simulation at 300 K. **(B)** Snapshots of the intermediate conformation of 2I9M, TC5B, 1WN8, 1V4Z, 1HO2, 1HLL, 2KFE, and 1YYB at various simulation times using MD simulation at 300 K. Here, the N terminal is always on the top.

To further illustrate the folding dynamic of for the eight studied proteins, conformational changes are monitored during AMD and MD simulations at 300 K and representative structures are shown in Figure 5. It is observed from the figure that the eight proteins are completely folded into their corresponding native structures in the AMD simulations. By contrast, all of the eight studied proteins fail to fold to their native structures in the traditional MD simulation.

Here, we discuss the conformational dynamics of each system in AMD simulation and traditional MD simulation in detail. After minimization and initial equilibration, all of the proteins maintain a considerable linear length. In AMD simulations, the folding paths of the different systems are different. We now briefly analyze the folding of each system.

Analysis of the results presented in Figure 5A provides a wealth of information regarding the folding of the eight studied proteins by AMD simulations. For 2I9M, the protein folds firstly from the region in the vicinity of the N-terminal at ~5 ns, and then the C-terminal residue begins to fold at 25 ns. Then, the protein gradually grows toward the center and the folding of the middle region is finished ~40 ns. For TC5B, the C-terminal residue folds firstly at ~10 ns and then the residues in the vicinity of the N-terminal fold at 35.6 ns. Finally, after 90 ns, the protein folds successfully. For 1WN8, the C-terminal region forms at 4 ns and the N-terminal starts to fold at 5 ns. The N-terminal and center are formed at 19 ns. Finally, the C-terminal is formed at 86 ns. For 1V4Z, the N-terminal starts to fold after 6 ns, folded into a helix structure from the N-terminal to the center at 18 ns, and finally the C-terminal folds at 43 ns. 1HO2 starts to fold from the residues close to the C-terminal at 2 ns, and then the residues fold toward the central region followed by the folding to the N-terminal and the C-terminal is formed at 180 ns. 1HLL firstly folds from the N-terminal at 8 ns and then the C-terminal starts to fold at 34 ns, with the N-terminal and C-terminal fully folds at 39 ns. Finally, the residues at the center completely fold in ~71 ns. The N-terminal of the 2KFE system begins to fold to form its helix structure after 3 ns and then the folding extends toward the center in ~16 ns. At 38 ns, the helix starts to form near the C-terminal and finally folds completely at 55 ns. The residue near the N-terminal of 1YYB begins to fold at 36 ns, and then the central region folds into a helix structure at 56 ns. Finally, the residue near the C-terminal begins to fold at 61 ns and the protein is completely folded by 64 ns. After all of the helices are completed, the RMSD values of the eight structures fluctuate ~0.71, 1.32, 0.60, 1.51, 1.27, 2.51, 2.12, and 1.28 Å, respectively, indicating that the peptides have reached a relatively stable state. Finally, we select the representative structure of 2I9M, TC5B, 1WN8, 1V4Z, 1HO2, 1HLL, 2KFE, and 1YYB in the AMD simulation at the final times of 51, 146, 146, 81, 196, 86, 61, and 86 ns, and these are found to be in good agreement with the corresponding native structures. It is observed that different systems have different folding paths. By contrast, an inspection of Figure 5B shows, that in all cases, the proteins do not fold successfully in the traditional MD simulations. Through the conformational changes in the graph, we find that there is no meaningful folding in the traditional MD simulation process. No protein is folded successfully from the linear structure to the corresponding native structure in traditional MD simulation during the same times as those used in the AMD simulations.

**Figure 6:**
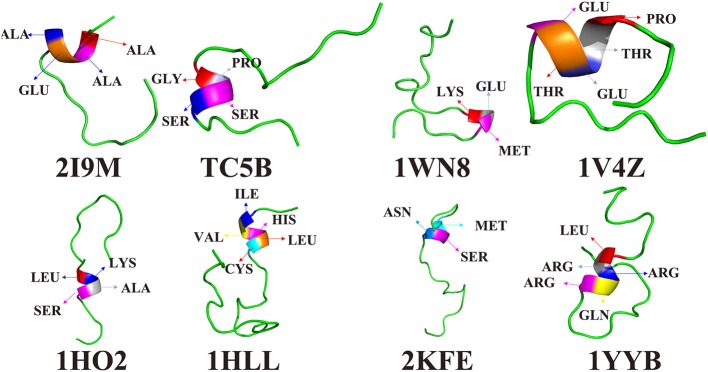
The residues of the first formed helix structures of 2I9M, TC5B, 1WN8, 1V4Z, 1HO2, 1HLL, 2KFE, and 1YYB by AMD simulation at 300 K.

To further understand the protein folding process, the initial folding residues of the eight proteins are shown in Figure 6. For 2I9M, the initial folded residue is ALA, ALA, GLU, and ALA. For TC5B, the GLY, PRO, SER, and SER residues initiate the folded. In the 1WN8, the first folded residues are LYS, GLU, and MET. For 1V4Z, the residues that first formed a small helix structure are PRO, THR, GLU, THR, and GLU. For 1HO2, the residues of the initial folded helix structure are LEU, LYS, ALA, and SER. For 1HLL, the original helical structure consists of five residues, namely, ILE, VAL HIS, LEU, and CYS. MET, SER, and ASN are the first to formed a helix structure in 2KFE. In 1YYB, the first helix structure consists of LEU, ARG, ARG, GLN, and ARG.

Interestingly, after studying all of the above residues that initially forms helical structures in the eight examined systems, we find no residues with a benzene ring structure. In other words, the probability that a residue containing benzene ring is initially folded into a helical structure is very low. It is likely that because benzene rings belong to rigid group, the residues containing benzene rings are not easy to fold. In the folding process, we find that residues near the initially formed structures fold faster than other residues. This maybe explains the origin of the different folding pathways observed for different proteins.

### Free Energy Landscape Analysis

**Figure 7:**
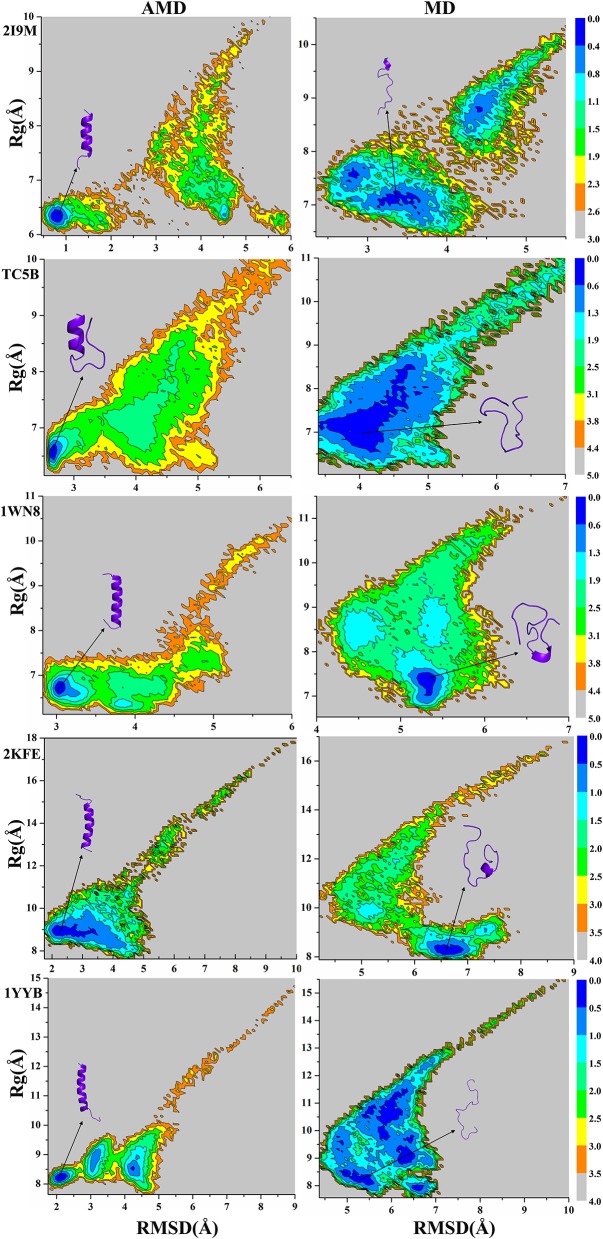
Free energy landscape as a function of RMSD and radius of gyration using AMD simulation and MD simulation for 2I9M, TC5B, 1WN8, 2KFE, and 1YYB during all the simulation time at 300 K, respectively. Those representative structures are also shown in the figure. The unit of free energy is kcal/mol.

Because the AMD method adds a robust bias potential energy to the actual potential energy, it is necessary to obtain the corrected canonical ensemble average of the system. Therefore, the strength of the Boltzmann factor of the bias energy at a particular point is used to reweight every point in the configuration space on the modified potential. Then the free energy landscape is calculated by the weighted histogram analysis method (WHAM) (Kumar et al., 1992, 1995) against the two reaction coordinates, the RMSD and the radius of gyration (R_g_). Here, we only analyze the free energy landscape sampled by the AMD and MD simulations at 300 K. Figure 7 not only provides the free energy landscape of five proteins (2I9M, TC5B, 1WN8, 2KFE, and 1YYB) simulated by AMD and MD methods, but also shows the representative structure in the lowest free energy state, providing a more intuitive understanding of the protein folding. The free energy diagram is drawn using the RMSD of the helix structure as the X-axis and *R*_g_ as the Y-axis.

From the free energy diagram, the lowest free energy states (LFES) of 2I9M, TC5B, 1WN8, 2KFE, and 1YYB are found at approximately (RMSD = 0.81 Å, *R*_g_ = 6.35 Å), (RMSD = 2.67 Å, *R*_g_ = 6.61 Å), (RMSD = 3.02 Å, *R*_g_ = 6.66 Å), (RMSD = 2.34 Å, *R*_g_ = 8.89 Å), and (RMSD = 2.11 Å, *R*_g_ = 8.17 Å) under the AMD simulation, respectively. This agrees quite well with their native structure. By contrast, the lowest energy conformation is located near (RMSD = 3.37 Å, *R*_g_ = 7.10 Å), (RMSD = 4.04 Å, *R*_g_ = 6.89 Å), (RMSD = 5.31 Å, *R*_g_ = 7.39 Å), (RMSD = 6.61 Å, *R*_g_ = 8.19 Å), and (RMSD = 5.35 Å, *R*_g_ = 8.18 Å) for the traditional MD simulation for the five proteins, respectively. Clearly, these states are far away from the corresponding native structures.

It is observed from the free energy landscape that there is no transition barrier on the surface of a single well in the AMD simulations, and that the folding of five proteins is a downhill folding process in a single state. Similarly, the structures extracted from the lowest energy states are consistent with the corresponding native structures, while the structures obtained in MD simulations are quite different. In conclusion, these results provide further validation of the superior efficiency and accuracy of AMD simulation relative to MD simulations.

To study whether the free energies of the five systems from the AMD simulation converge, we create three free energy landscapes for the total equilibrium time, the first half of the equilibrium time and the second half of the equilibrium time, are shown in Figures S1–S3 in the Supporting Information, respectively. Table S3 in the Supporting Information shows the values of RMSD and *R*_g_ corresponding to the lowest energy states of five proteins at different time periods.

It is observed from Figures S1–S3 and Table S3 that there are slight differences in the free energy for the three time periods of five proteins in AMD simulations. The important thing is that these structures extracted from the lowest energy states are all coincide with the correspond native structure. However, the free energy landscapes of all of the systems in the three periods show larger differences in MD simulations. It is clear that free energy convergence can be achieved by using the AMD method in each system, while free energy convergence cannot be achieved in MD simulations during the same time.

### Radius of Gyration (*R*_g_)

**Figure 8:**
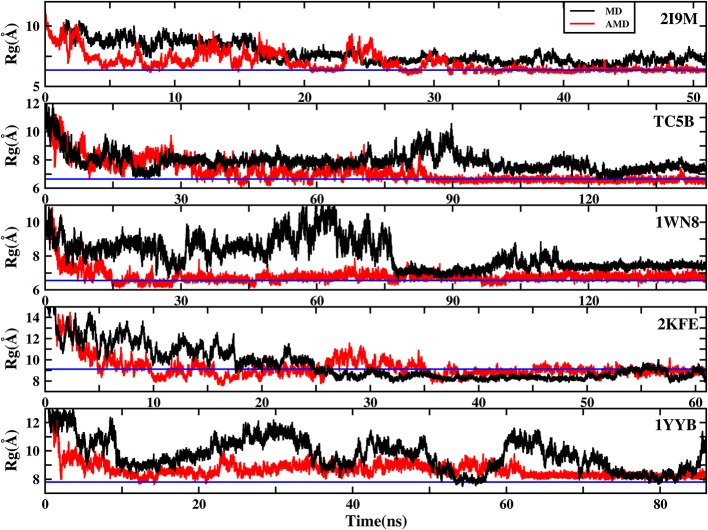
The radius of gyration (*R*g) of the helix structure as a function of simulation time using the AMD simulation (red) and MD simulation (black) at 300 K for 2I9M, TC5B, 1WN8, 2KFE, and 1YYB, respectively. Blue lines denote those experimental values.

To further understand the evolution of other properties of the protein during the folding from linear state to the native structure, we analyze the evolution of the radius of gyration of the above five proteins during AMD simulations and during MD simulation at 300 K. Figure 8 shows that the folding process of the five systems of AMD simulations can be roughly divided into three stages. The initial R_g_s values are 11.34, 12.48, 12.63, 18.16, and 16.45 Å, respectively, which are the maximum value for the five proteins. In the first stage, all of the proteins are just starting to fold and *R*_g_s values rapidly collapse to ~7.00, 6.70, 6.20, 8.00, and 7.70 Å at ~5.0, 17.0, 15.0, 10.0, and 12.50 ns for the five proteins, respectively. In the second stage, the *R*_g_s values of the five systems have large fluctuations in a broad region (between 6 and 9.5 Å), (between 6 and 10 Å), (between 6 and 8 Å), (between 8 and 11 Å), and (between 8 and 10 Å) until ~35, 85, 100, 45, and 62 ns, respectively. In the final stage, the R_g_s values of the five proteins fluctuate near the corresponding experimental values (6.34, 6.64, 6.55, 9.12, and 7.79 Å), respectively, suggesting that the AMD simulations have converged and the folding has been completed successfully. However, the *R*_g_s values obtained in MD simulation in these systems are greater than their experimental values, indicating that MD simulations failed to fold the proteins into their native structures.

### The Effect of Different High Temperature

#### Cluster Analysis

**Figure 9:**
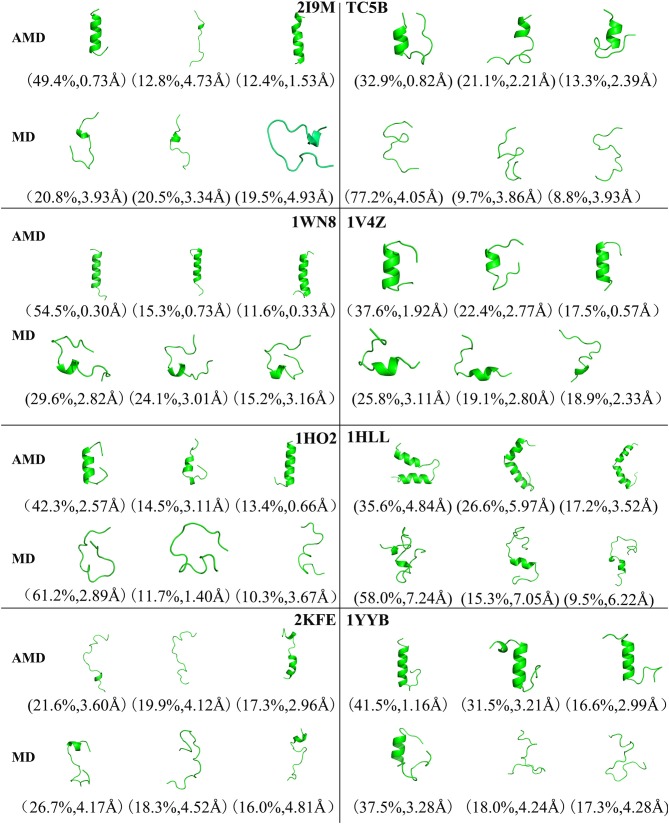
Representative structures of 2I9M, TC5B, 1WN8, 1V4Z, 1HO2, 1HLL, 2KFE, and 1YYB conformations selected from the top three occupied clusters using AMD simulation (top) and MD simulation (low) at 350 K. The population of clusters and the backbone RMSD of the cluster centers are indicated.

**Figure 10:**
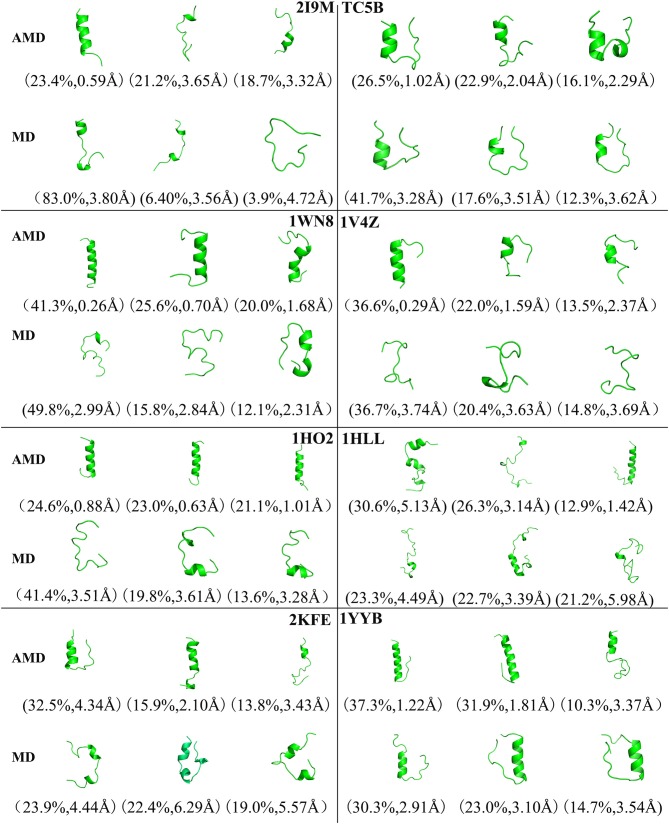
Representative structures of 2I9M, TC5B, 1WN8, 1V4Z, 1HO2, 1HLL, 2KFE, and 1YYB conformations selected from the top three occupied clusters using AMD simulation (top) and MD simulation (low) at 400 K. The population of clusters and the backbone RMSD of the cluster centers are indicated.

**Figure 11:**
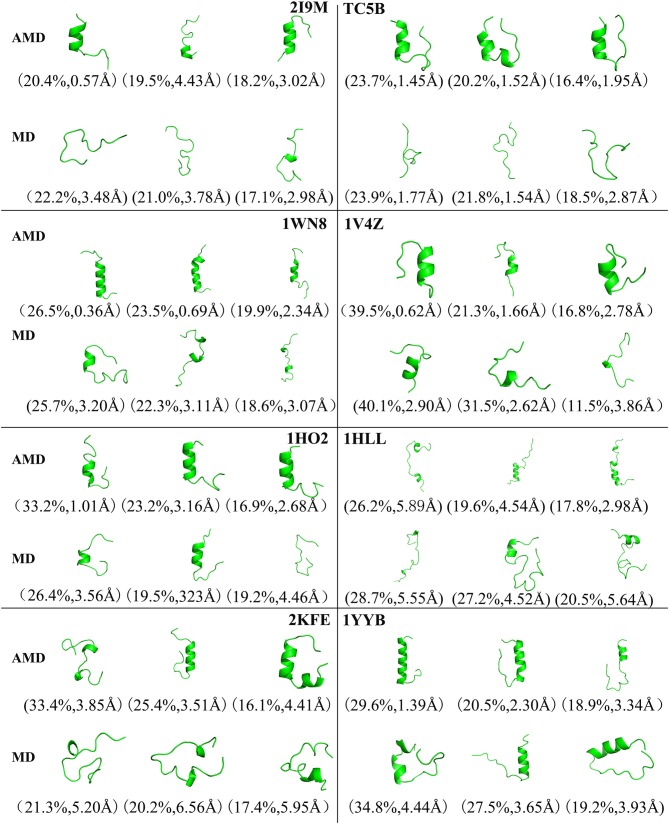
Representative structures of 2I9M, TC5B, 1WN8, 1V4Z, 1HO2, 1HLL, 2KFE, and 1YYB conformations selected from the top three occupied clusters using AMD simulation (top) and MD simulation (low) at 450 K. The population of clusters and the backbone RMSD of the cluster centers are indicated.

To analysis the difference between the AMD and MD simulation, we carry out clustering analysis at different temperatures. Firstly, we compare the differences between AMD simulation and MD simulation at the same temperature. Figure 9 shows the representative structures of the top three occupied clusters for the eight proteins studied in this work during the last 20 ns of the simulation process at 350 K. The fraction of RMSD values <1 Å are 49.4, 32.9, 54.5, 17.5, 13.4% by AMD simulation while their fractions are 0, 0, 0, 0, and 0% by MD simulation in the system of 2I9M, TC5B, 1WN8, 1V4Z, and 1HO2. At the same time, the RMSD values of the first cluster in the five systems in the AMD simulation are all lower than those in the MD simulation. In the system of 1HLL, 2KFE, 1YYB, although the RMSD values of both AMD and MD are large, AMD is still smaller than MD. Figure 10 shows the responding result at 400 K. We can see that the fraction of RMSD values <1 Å are 23.4, 66.9, 36.6, 47.6% in AMD simulation at 400 K, while their fractions are all 0% under traditional MD simulation in the system of 2I9M, 1WN8, 1V4Z, and 1HO2. In AMD simulation, RMSD values of the first three clusters in TC5B are all <2.3 Å, while the RMSD values in MD simulation are all more than 3.2 Å. The fraction of RMSD value <1.5 Å in 1HLL system is 12.9% by AMD simulation, while the RMSD value in all cluster in MD simulation is more than 3.3 Å. In the 2KFE system, the values of the RMSD of the top three clusters by AMD simulation are lower than MD simulation. The ratio of the value of RMSD by AMD simulation less 2 Å is 62.9%, while all RMSD values in MD simulation are >2.9 Å. Figure 11 shows the result at 450 K. From Figure 11, the proportion of the RMSD values by AMD simulation under 1 Å is 20.4, 26.5, and 39.5% in the system of 2I9M, 1WN8, and 1V4Z, while the value of the RMSD using MD simulation is all >2.9 Å. The value of the RMSD of the top three cluster by AMD simulation is much lower than correspondence structure in the MD simulation in the system of TC5B, 1HO2, 2KFE, and 1YYB. The proportion of the value of the RMSD by AMD simulation under 3 Å is 17.8%, while the value of the RMSD using MD simulation is all >4.5 Å.

From Figures 4, 9–11, we compare the effects of the temperature on each system in the AMD simulations. We can see very clearly that 300 K has the smallest RMSD values with the highest percentage in the four temperatures. In other words, the proportion of structure of native or close to native state is the highest by AMD simulation at 300 K. We can also see that the 2KFE system has the native state structure at 300 K, and no folding state is found in the top three cluster at three high temperatures. To summarize, we can conclude that the efficiency of AMD simulations at different temperatures is higher than that of MD simulations, and different systems show different temperature sensitivities; however, 300 K is generally the most favorable temperature for protein folding. In addition, temperature has the most significant effect on the system of 2KFE.

#### Helix Content and Root Mean Square Deviation (RMSD) Analysis

Next, Figures S4–S6 in the Supporting Information illustrates the detailed development about the fractional native helix content during the last 20 ns of the AMD simulation and MD simulation at different temperature (350, 400, 450 K) for eight proteins, respectively. It is clear that the overall fractional native helix contents in AMD simulation are higher than that in MD simulation, which is in excellent agreement with the above analysis.

To study the effect of the temperature on system simulation, the backbone RMSD distributions during the last 20 ns of the AMD and MD simulation at different temperatures (350, 400, 450 K) are shown in Figures S7–S9 in the Supporting Information for eight systems. We can clearly see that in the AMD simulation the values of the RMSD of the most populated states of all systems at all temperatures is smaller than that in MD simulation, except for the system of 2I9M at 450 K temperature. In 2I9M system, although there is no significant difference in the most probable distribution of RMSD values between MD and AMD simulation. In the AMD simulation, these structures with RMSD <1 Å are more than MD simulation. In addition, all of the RMSD distributions in AMD are trend to the smaller values than in MD simulations. The result indicates that the AMD simulation is highly efficient for studying the folding of proteins.

### Multiple-Trajectory Result

**Figure 12:**
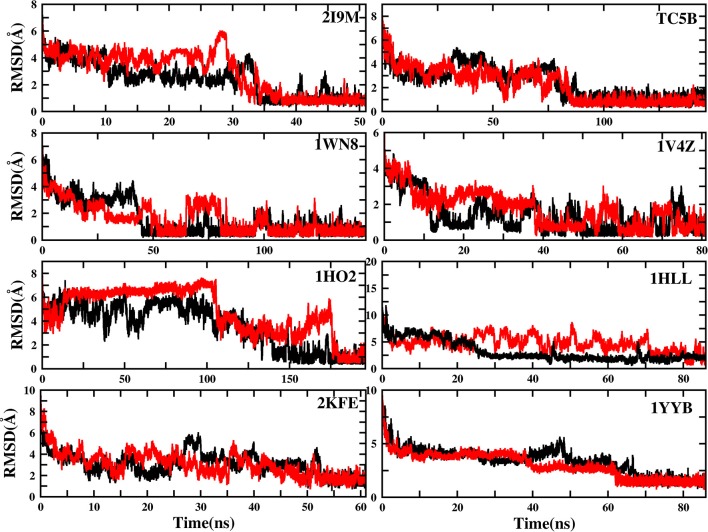
RMSD of backbone atoms of 2I9M, TC5B, 1WN8, 1V4Z, 1HO2, 1HLL, 2KFE, and 1YYB as a function of MD simulation time from two MD trajectories. The red curve denotes the trajectory discussed in the current paper and the black curve denotes another trajectory with the same starting structure but different random seed for momentum.

It should be noted here that the results based on a single AMD simulation trajectory may not be sufficient to support the conclusion that AMD simulations accelerate protein folding. So, we perform another set of independent AMD simulation of the eight proteins starting from the linear initial structure at 300 K. Figure 12 shows the plots of the two trajectories for eight different systems, and the evolution of RMSD value over time is analyzed. As observed from Figure 12, the RMSD values of the eight systems in the new trajectories fluctuate ~0.81, 1.31, 0.56, 0.38, 1.39, 2.39, 1.99, and 1.31 Å in ~34, 80, 45, 40, 150, 28, 53, and 68 ns, respectively. In addition, the minimum RMSD values of the eight systems are ~0.35 Å, 0.33, 0.22, 0.18, 0.35, 0.63, 0.61, and 0.83 Å, respectively. It is observed from the figure that although the details of the folding paths and steps for each individual trajectory are not exactly the same, for both trajectories, the proteins fold into the corrected native structures by AMD simulation.

## Conclusion

In this work, we perform *ab* initio folding for eight proteins (2I9M, TC5B, 1WN8, 1V4Z, 1HO2, 1HLL, 2KFE, and 1YYB) using AMD simulations and traditional MD simulation at different temperatures (300, 350, 400, and 450 K) in explicit water. The advantages of the AMD approach over the traditional MD are as follows: First, AMD provides efficient enhanced conformational space sampling. Second, it does not require the knowledge of the free energy surface in advance and does not need to define the reaction coordinates in advance.

The results of the AMD and MD simulations of the eight systems at 300 K are analyzed and compared using the following six aspects: the RMSD, the native contacts, the cluster analysis, the process of the protein folding, the *R*_g_ and the free energy landscape. The final RMSD values are 0.77, 0.65, 0.06, 0.98, 1.10, 1.93, 1.49, and 1.51 Å in AMD simulation, while these values are 2.69, 4.72, 4.19, 3.531, 6.56, 7.17, 6.13, and 4.94 Å for the MD simulation, respectively. The native contacts analysis shows that the fraction of the native contacts of these eight proteins in AMD simulation are clearly higher than those in the MD simulation, indicating that the structures generated from AMD simulations are more consistent with the native states. Cluster analysis shows that the conformations in the native state cluster have the highest occupancy for AMD simulations at 300 K. Then, the folding pathways are further discussed and analyzed for the eight proteins using AMD simulations at 300 K. Although these proteins have different folding pathways, they all finally fold into their native structures. Free energy landscape analysis shows that the structures in the lowest free energy state in AMD simulation at 300 K are the native structures. *R*_g_s values fluctuate around their corresponding native values suggesting that these folding simulations are generally completed. The above described results show that AMD simulations can correctly fold the proteins into native structures, but the same protein folding fail when the traditional MD simulations are carried out for the same times as the AMD simulation at 300 K.

Based on the cluster analysis, the native helix content analysis and RMSD distribution analysis of the AMD and MD results at different high temperatures (350, 400, 450 K), we find that the simulation efficiency of AMD is higher than that of MD at all temperatures. It was also found that 300 K is the most suitable temperature for the folding of all proteins.

In this report, AMD is used to fold helical protein in explicit water. We examine the applicability of AMD simulation in folding by testing a series of proteins and find that it has the advantages of fast speed and small calculation. The efficiency and accuracy of the AMD simulation method compared with the traditional MD simulation method are verified. Generally, the AMD simulation results obtained here are very encouraging for the further use of this method in the studies the protein folding. Our studies on the folding of these eight proteins will provide useful guidance for other protein folding investigations.

## Author Contributions

LD designed this study and revised the manuscript. YC and XG outperformed the MD simulations and prepared all the figures. GF, YL, and JZ helped with data analysis.

### Conflict of Interest Statement

The authors declare that the research was conducted in the absence of any commercial or financial relationships that could be construed as a potential conflict of interest.
