# Supplementary material for: Accelerated Molecular Dynamics Simulation for Helical Proteins Folding in Explicit Water
Source: Front Chem. 2019 Aug 6;7:540. doi: 10.3389/fchem.2019.00540 (PMC6691143; doi:10.3389/fchem.2019.00540)
Supplement: Supplementary file 1 [file Data_Sheet_1.pdf]

# Supporting Information

# Accelerated molecular dynamics simulation for helical proteins folding in explicit water

Lili Duan,*[,1] Xiaona Guo,[1] Yalong Cong,[2] Guoqiang Feng,[1] Yuchen Li,[1] John Z. H. Zhang, [2,3,4]

[1]School of Physics and Electronics, Shandong Normal University, Jinan 250014, China

[2]Shanghai Engineering Research Center of Molecular Therapeutics and New Drug Development, School of Chemistry and Molecular Engineering, East China Normal University, Shanghai, 200062, China

[3]NYU-ECNU Center for Computational Chemistry at NYU Shanghai, Shanghai 200062, China

[4]Department of Chemistry, New York University, NY, NY 10003, USA

Corresponding author (duanll@sdnu.edu.cn)

**Table S1.** Summary of the parameters of the AMD simulations at different temperature for 2I9M, TC5B, 1WN8, 1V4Z, 1HO2, 1HLL, 2KFE and 1YYB, respectively.

**Table S2.** The corresponding RMSD values at different times in the AMD simulation at 300 K for 2I9M, TC5B, 1WN8, 1V4Z, 1HO2, 1HLL, 2KFE and 1YYB, respectively.

**Table S3.** The values of the RMSD and $R_g$ during different equilibrium periods in AMD simulation and MD simulation at 300 K for 2I9M, TC5B, 1WN8, 2KFE and 1YYB, respectively.

**Table S1**

| PDB | 300K ($E_D$, $\alpha_D$, $E_P$, $\alpha_P$) | 350K ($E_D$, $\alpha_D$, $E_P$, $\alpha_P$) | 400K ($E_D$, $\alpha_D$, $E_P$, $\alpha_P$) | 450K ($E_D$, $\alpha_D$, $E_P$, $\alpha_P$) |
|---|---|---|---|---|
| **2I9M** | 220.3, 11.9, -99701.5, 6704 | 227.7, 11.9, -924788.1, 6704 | 234.7,11.9, -85278.7, 6704 | 241.2, 11.9, -77664.6, 6704 |
| **TC5B** | 311.74, 14, -124200.26, 8359 | 320.3, 14, -115203.5, 8359 | 328.9, 14, -106179.1, 8359 | 332.5, 14, -96740.7, 8359 |
| **1WN8** | 339.05, 15.4, -184571.5, 12423.2 | 344.5, 15.4, -171218.5, 12423.2 | 350,15.4, -157836.8,12423.2 | 359,15.4, -143749,12423.2 |
| **1V4Z** | 260.55, 11.9, -93204.99, 6265.2 | 269.7, 11.9, -86446.2, 6265.2 | 275.6, 11.9 -79695.2, 6265.2 | 280.5,11.9, -72597.1, 6265.2 |
| **1HO2** | 284.35, 14, -151234.44, 10150 | 293.2,14, -140314.3, 10150 | 300.1, 14, -129425.6 ,10150 | 304.5, 14, -117897.8, 10150 |
| **1HLL** | 511.78, 22.4, -388232.11, 31251.4 | 525.4, 22.4, -425865.3, 31251.4 | 541,22.4, -391266.3,31251.4 | 554,22.4, -354210, 31251.4 |
| **2KFE** | 354.1, 16.8, -214129.75, 14363 | 362.5, 16.8, -198703.2, 14363 | 375, 16.8, -183220.5, 14363 | 387, 16.8, -166919.3, 14363 |
| **1YYB** | 399.04, 18.2, -244550.67,16387.2 | 406.5, 18.2, -226931.2, 16387.2 | 417.7, 18.2, -209295, 16387.2 | 424,18.2, -190659, 16387.2 |

**Table S2**

| PDB | Initial value | First helical structure formed | | Stable helical structure formed | | Finally | |
|---|---|---|---|---|---|---|---|
| | RMSD | Time | RMSD | Time | RMSD | Time | RMSD |
| 2I9M | 6.92 | 5.60 | 4.37 | 40.20 | 0.70 | 51.00 | 0.77 |
| TC5B | 8.36 | 10.00 | 3.96 | 90.00 | 1.30 | 146.00 | 0.65 |
| 1WN8 | 7.25 | 4.64 | 4.20 | 86.00 | 0.60 | 146.00 | 0.66 |
| 1V4Z | 6.11 | 6.20 | 3.80 | 43.00 | 1.65 | 81.00 | 0.98 |
| 1HO2 | 8.79 | 2.00 | 4.47 | 180.00 | 1.20 | 196.00 | 1.10 |
| 1HLL | 11.68 | 8.00 | 4.21 | 71.60 | 2.50 | 86.00 | 1.93 |
| 2KFE | 9.98 | 3.40 | 4.04 | 55.20 | 2.10 | 61.00 | 1.49 |
| 1YYB | 10.80 | 36.40 | 3.76 | 64.20 | 1.20 | 86.00 | 1.51 |

**Table S3**

| PDB | Time (ns) | RMSD(Å) | Rg(Å) |
|---|---|---|---|
| 2I9M | 36~51 | 0.79 | 6.43 |
| | 36~46 | 0.75 | 6.39 |
| | 46~51 | 0.76 | 6.31 |
| TC5B | 86~146 | 0.62 | 6.53 |
| | 86~116 | 0.66 | 6.57 |
| | 116~146 | 0.63 | 6.52 |
| 1WN8 | 86~146 | 0.52 | 6.70 |
| | 86~116 | 0.51 | 6.74 |
| | 116~146 | 0.52 | 6.71 |
| 2KFE | 51~61 | 1.89 | 8.77 |
| | 51~56 | 1.87 | 8.91 |
| | 56~61 | 2.09 | 8.91 |
| 1YYB | 61~86 | 1.43 | 8.19 |
| | 61~76 | 1.49 | 8.21 |
| | 76~86 | 1.49 | 8.28 |

**Figure S1.** Free energy landscape as a function of RMSD and radius of gyration using AMD simulation and MD simulation for 2I9M (36~51 ns), TC5B (86~146 ns), 1WN8 (86~146 ns), 2KFE (51~61 ns) and 1YYB (61~86 ns) in the equilibrium time at 300 K, respectively. Those representative structures are also shown in the following figure. The unit of free energy is kcal/mol.

**Figure S2.** Free energy landscape as a function of RMSD and radius of gyration using AMD simulation and MD simulation for 2I9M (36~46 ns), TC5B (86~116 ns), 1WN8 (86~116 ns), 2KFE (51~56 ns) and 1YYB (61~76 ns) in the first half of the equilibrium time at 300 K, respectively. Those representative structures are also shown in the following figure. The unit of free energy is kcal/mol.

**Figure S3.** Free energy landscape as a function of RMSD and radius of gyration using AMD simulation and MD simulation for 2I9M (46~51 ns), TC5B (116~146 ns), 1WN8 (116~146 ns), 2KFE (56~61 ns) and 1YYB (76~86 ns) in the second half of the equilibrium time at 300 K, respectively. Those representative structures are also shown in the following figure. The unit of free energy is kcal/mol.

**Figure S4.** Fractional native helix content during AMD and MD simulation as a function of time using explicit solvent model at 350 K for 2I9M, 1WN8, 1V4Z, 1HO2, 1HLL, 2KFE and 1YYB, respectively.

**Figure S5.** Fractional native helix content during AMD and MD simulation as a function of time using explicit solvent model at 400 K for 2I9M, 1WN8, 1V4Z, 1HO2, 1HLL, 2KFE and 1YYB, respectively

**Figure S6.** Fractional native helix content during of the AMD and MD simulation as a function of time using explicit solvent model at 450 K for 2I9M, 1WN8, 1V4Z, 1HO2, 1HLL, 2KFE and 1YYB, respectively

**Figure S7.** RMSD of backbone atoms as a function of the AMD and MD simulation time under explicit solvent model at 350 K for 2I9M, TC5B, 1WN8, 1V4Z, 1HO2, 1HLL, 2KFE and 1YYB, respectively.

**Figure S8.** RMSD of backbone atoms as a function of the AMD and MD simulation time under explicit solvent model at 400 K for 2I9M, TC5B, 1WN8, 1V4Z, 1HO2, 1HLL, 2KFE and 1YYB, respectively.

**Figure S9.** RMSD of backbone atoms as a function of the AMD and MD simulation time under explicit solvent model at 450 K for 2I9M, TC5B, 1WN8, 1V4Z, 1HO2, 1HLL, 2KFE and 1YYB, respectively.

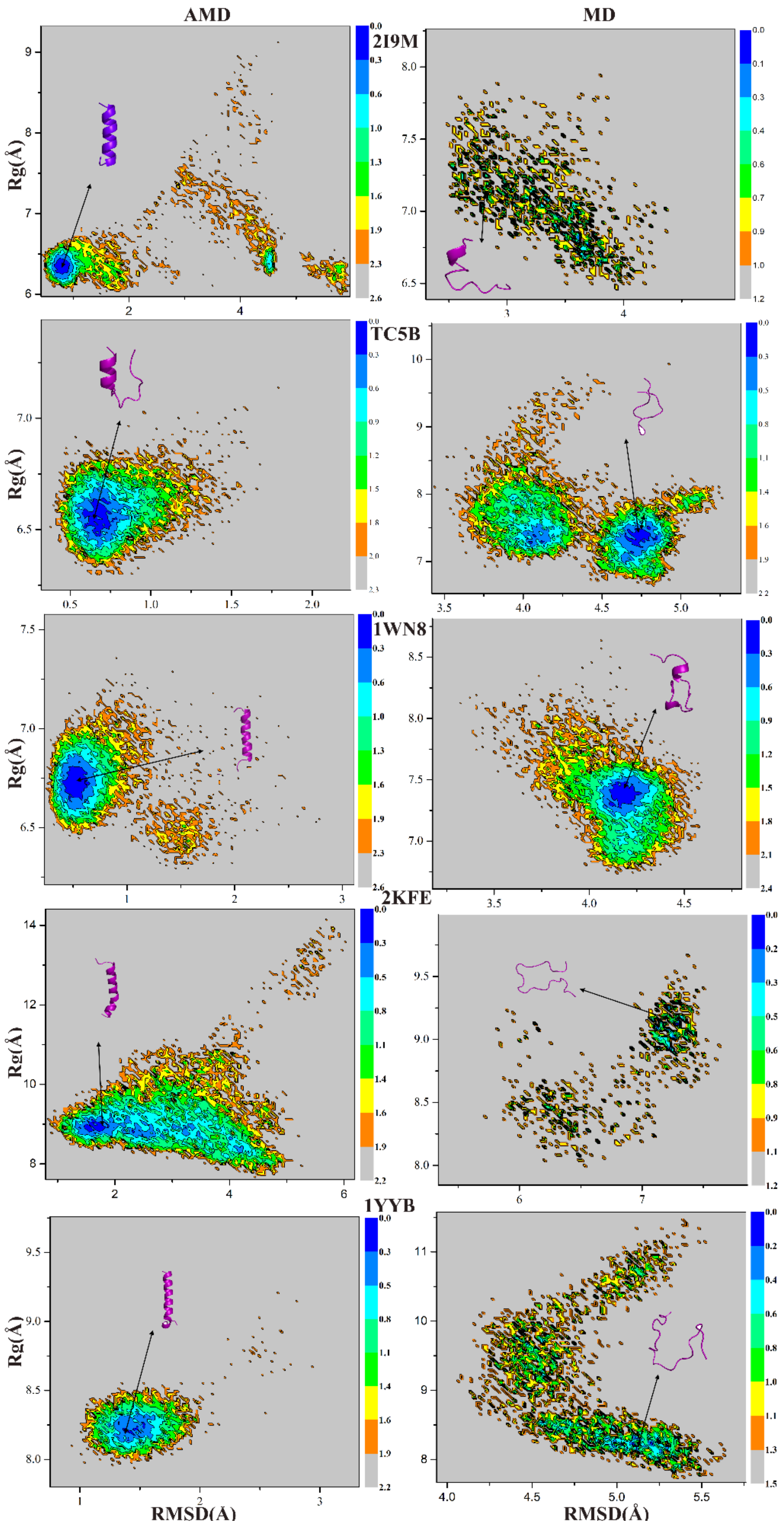


Figure S1

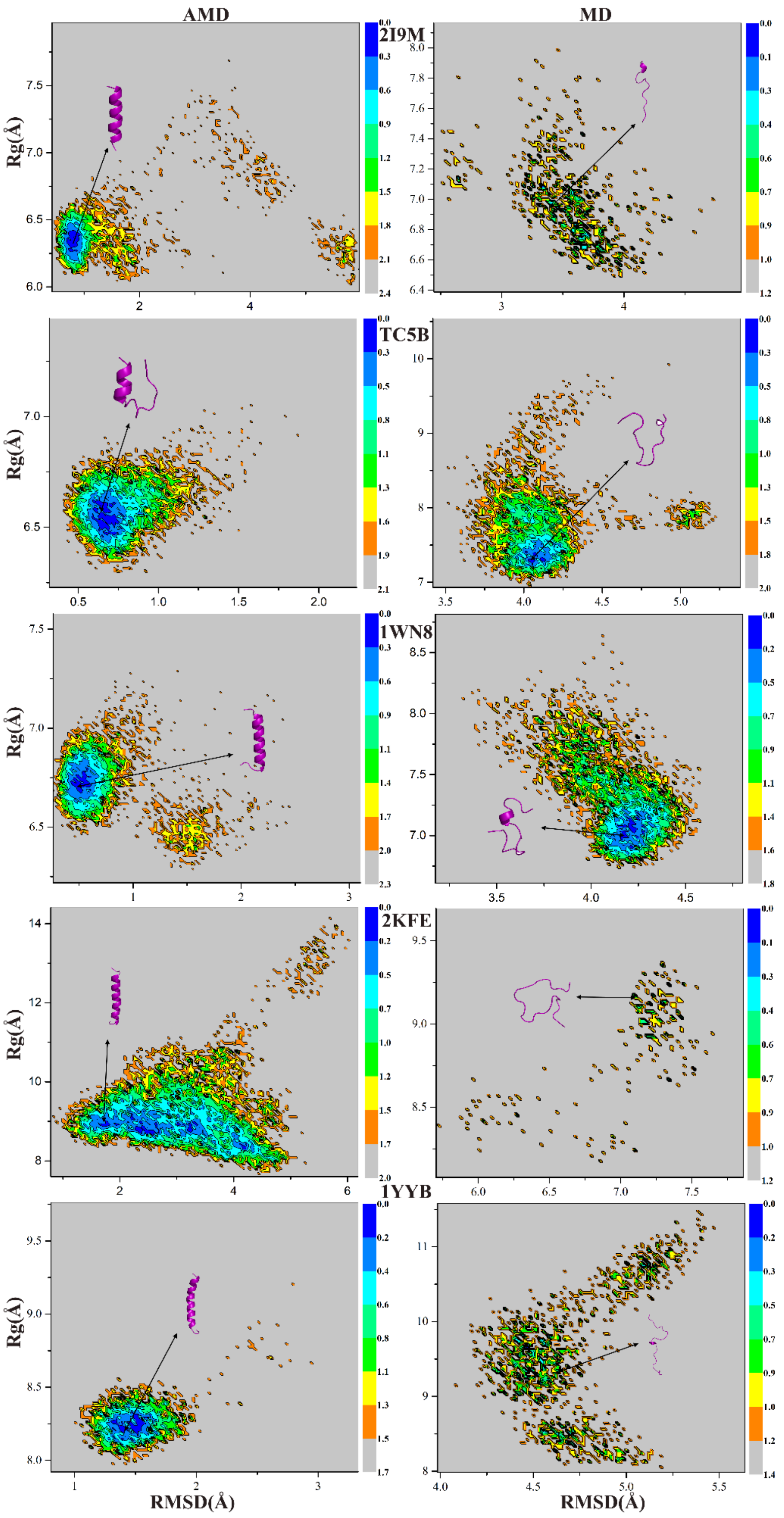


Figure S2

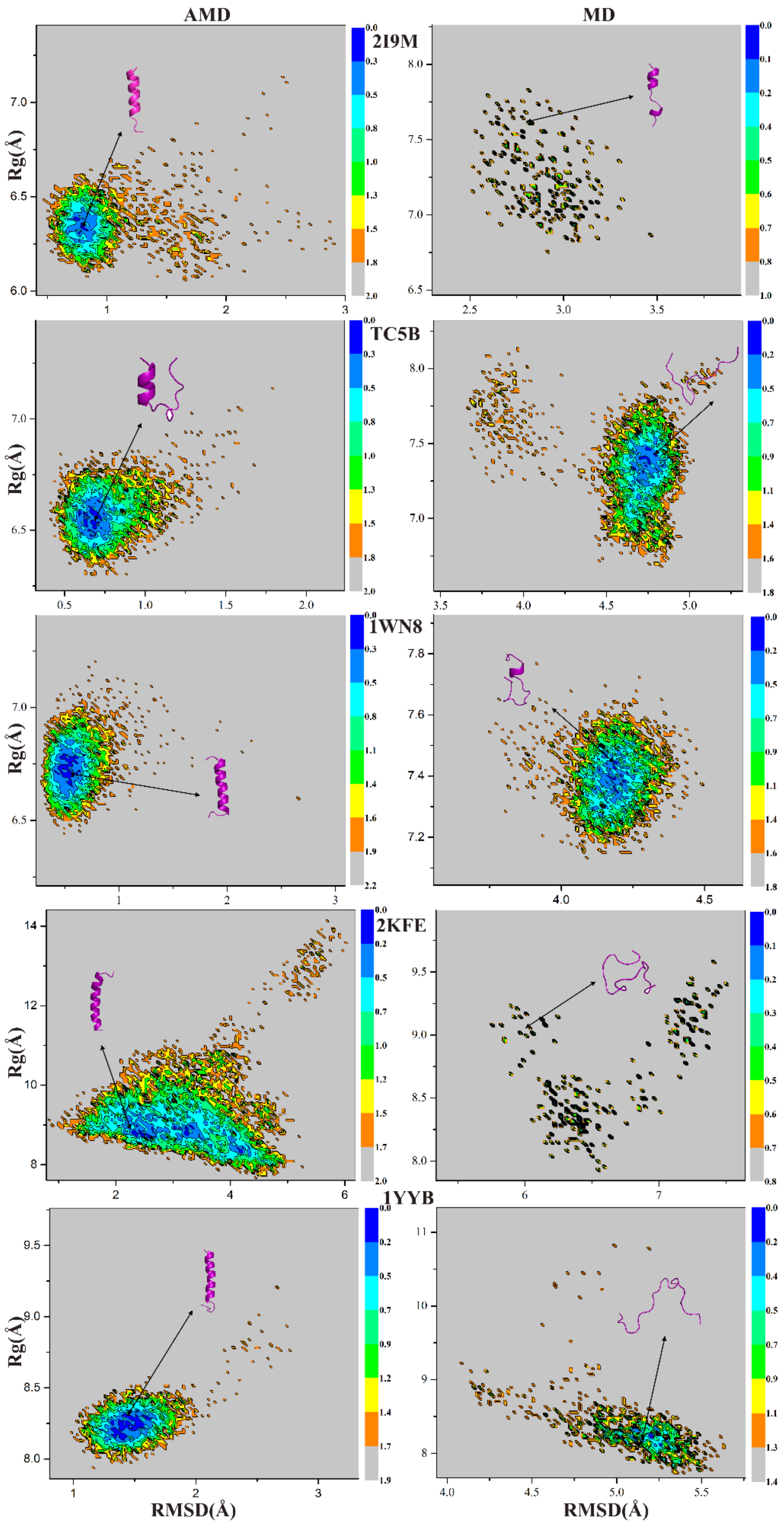


Figure S3

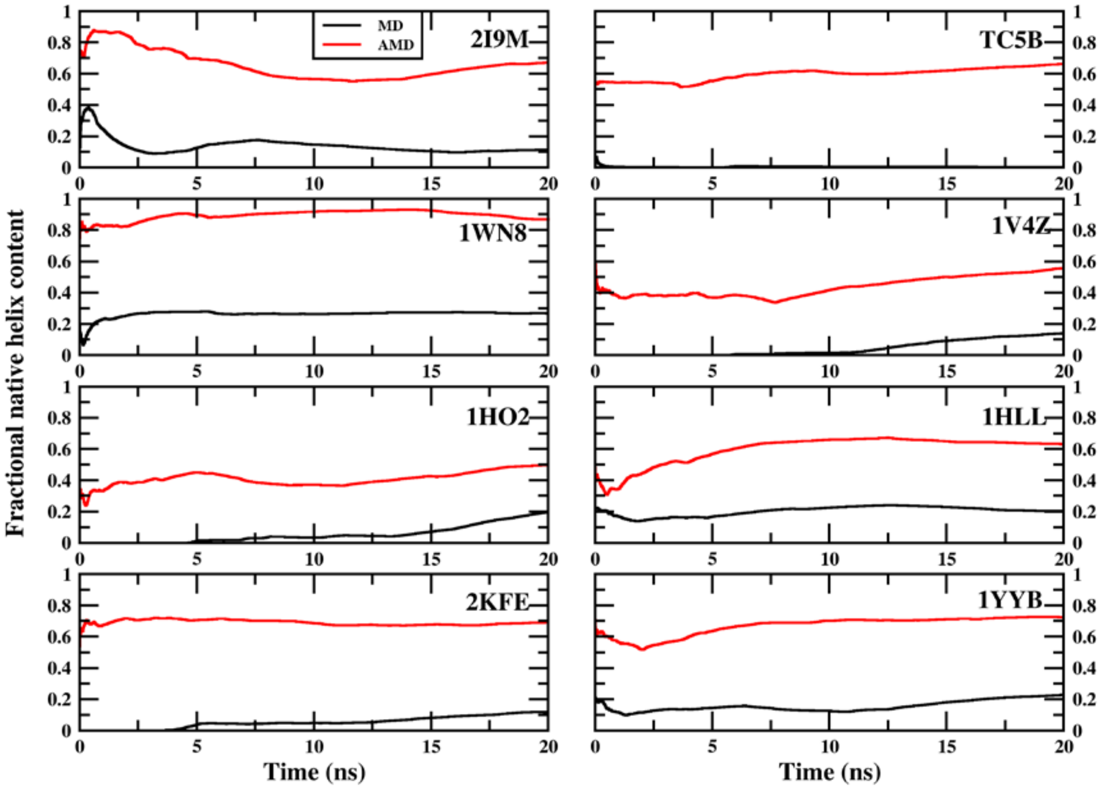


Figure S4

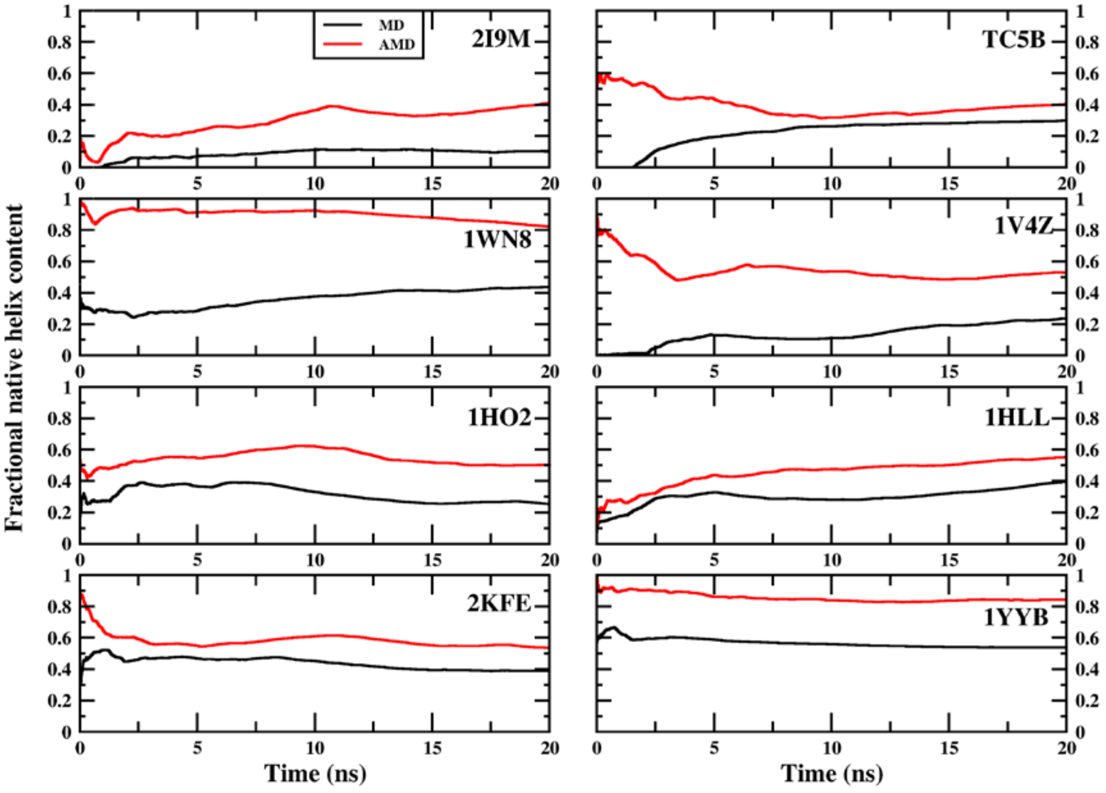


Figure S5

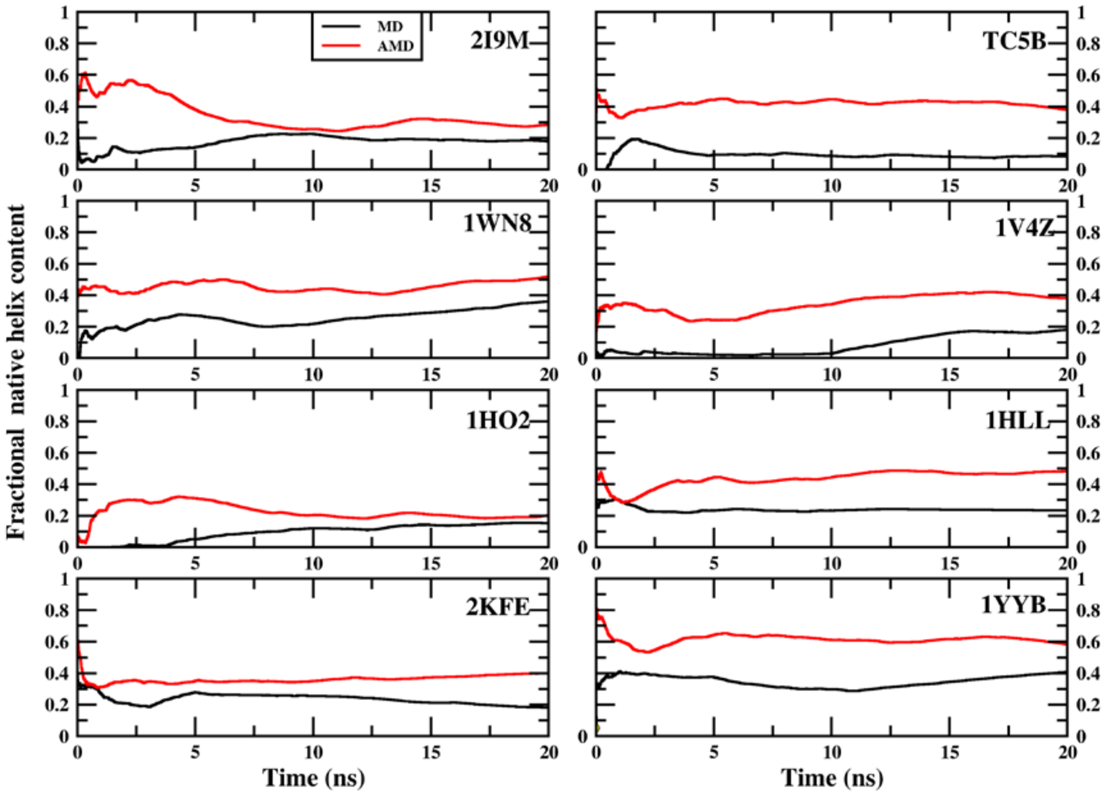


Figure S6

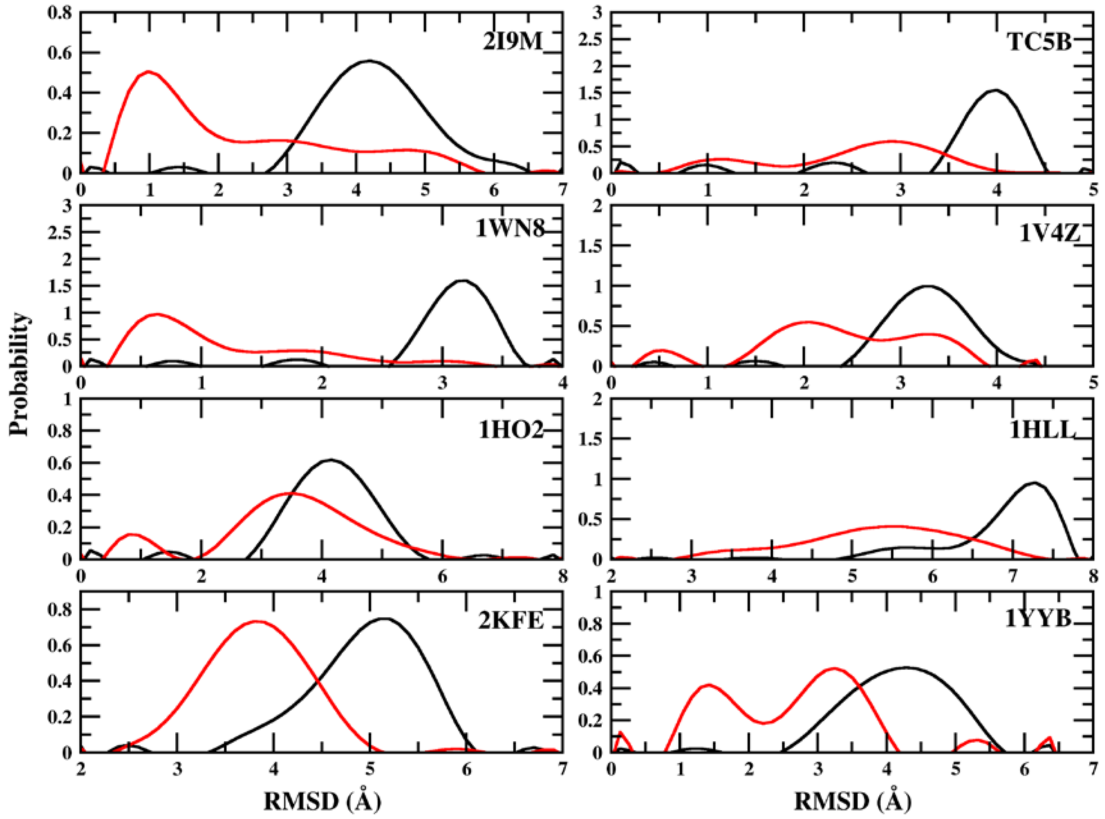


Figure S7

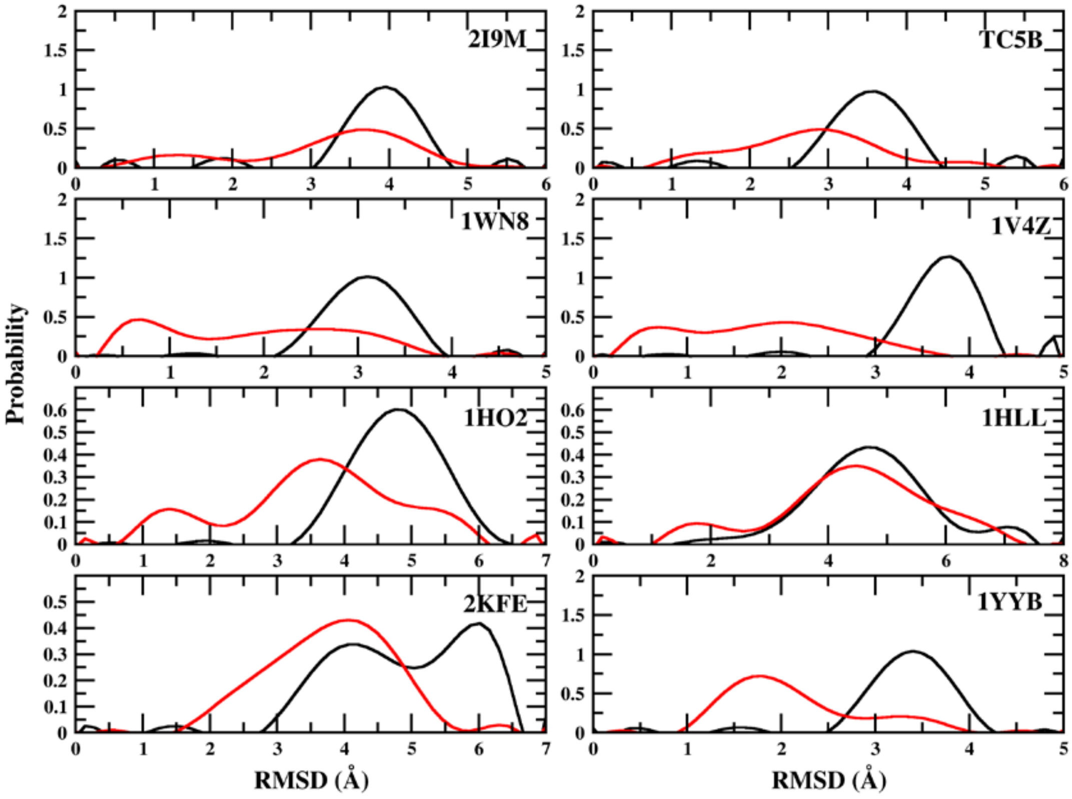


Figure S8

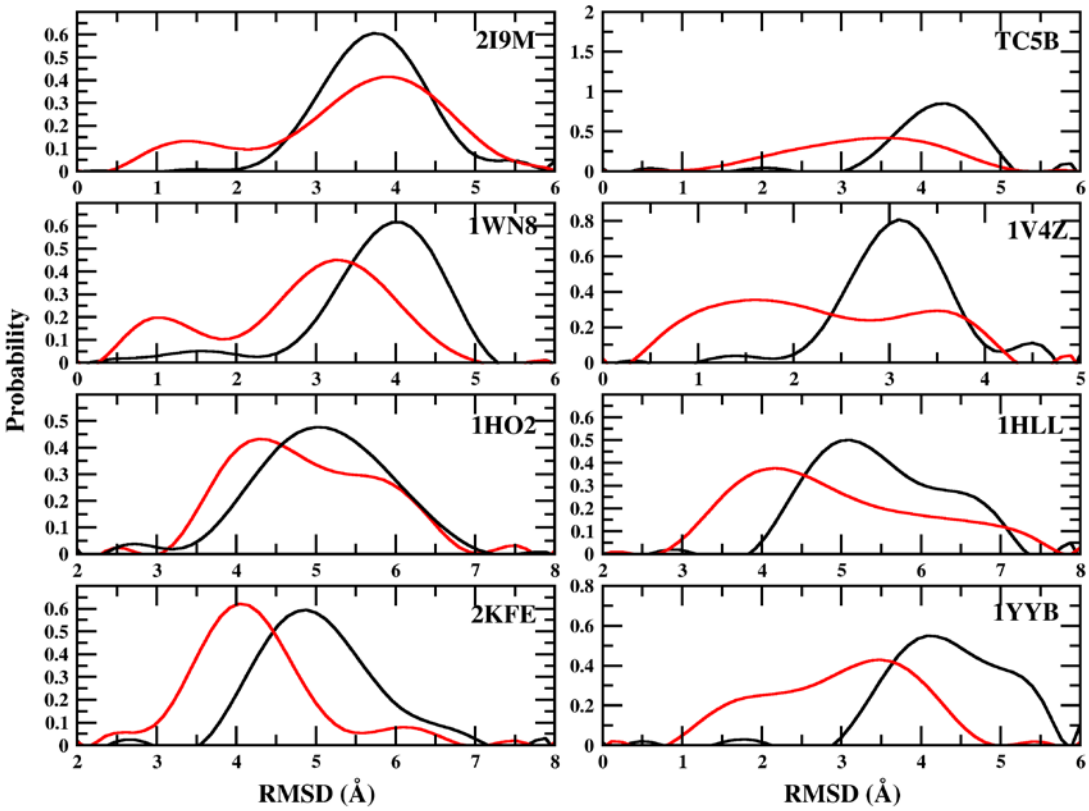


Figure S9
